# A cluster of *Candida krusei *infections in a haematological unit

**DOI:** 10.1186/1471-2334-7-97

**Published:** 2007-08-22

**Authors:** Timo Hautala, Irma Ikäheimo, Heidi Husu, Marjaana Säily, Timo Siitonen, Pirjo Koistinen, Jaana Vuopio-Varkila, Markku Koskela, Pekka Kujala

**Affiliations:** 1 Department of Internal Medicine, Oulu University Hospital, Oulu, Finland; 2 Clinical Microbiology Laboratory, Oulu University Hospital, Oulu, Finland; 3 Department of Medical Microbiology, University of Oulu, Oulu, Finland; 4 Department of Bacterial and Inflammatory Diseases, National Public Health Institute, Helsinki, Finland

## Abstract

**Background:**

*Candida krusei *infections are associated with high mortality. In order to explore ways to prevent these infections, we investigated potential routes for nosocomial spread and possible clonality of *C. krusei *in a haematological unit which had experienced an unusually high incidence of cases.

**Methods:**

We searched for *C. krusei *contamination of the hospital environment and determined the level of colonization in patients and health care workers. We also analyzed the possible association between exposure to prophylactic antifungals or chemotherapeutic agents and occurrence of *C. krusei*. The *C. krusei *isolates found were genotyped by pulsed-field electrophoresis method in order to determine possible relatedness of the cases.

**Results:**

Twelve patients with invasive *C. krusei *infection and ten patients with potentially significant infection or mucosal colonization were documented within nine months. We were unable to identify any exogenic source of infection or colonization. Genetic analysis of the isolates showed little evidence of clonal transmission of *C. krusei *strains between the patients. Instead, each patient was colonized or infected by several different closely related genotypes. No association between medications and occurrence of *C. krusei *was found.

**Conclusion:**

Little evidence of nosocomial spread of a single *C. krusei *clone was found. The outbreak may have been controlled by cessation of prophylactic antifungals and by intensifying infection control measures, e.g. hand hygiene and cohorting of the patients, although no clear association with these factors was demonstrated.

## Background

*Candida *infections are common in immunocompromized host and result in significant mortality and extended hospital treatement [1-3]. An increase in prevalence of species of *Candida *other than *C. albicans*, such as *Candida glabrata *and *Candida krusei*, has been recognized in some hospitals while they are still rare in many other institutions [4-10]. A number of fungal pathogens are inherently resistant to fluconazole and amphotericin B and require alternative antifungals, such as echinocandins or the newer azoles. It is speculated that use of these agents may in turn contribute to further selection pressure towards these fungi and may therefore partially explain, for example, an increase in the occurrence of zygomycoses. Invasive infections or colonization with *C. krusei *has been associated with prophylactic use of antifungals [4,11,12]. It is thought that suppression of common *Candida *species with fluconazole prophylaxis may allow the growth of less pathogenic and inherently resistant or less susceptible species of *Candida*. The role of the prophylactic fluconazole, however, has not been evident in all outbreak settings and other antimicrobial agents may contribute to colonization with the species of *Candida *other than *C. albicans *[13-15].

Clonal spread of *C. albicans *has been verified in a number of outbreak situations [7,16,17]. *Candida parapsilosis *is a common finding on healthy hands and clonal nosocomial spread on the hands of health care workers has been demonstrated [18,19]. There is some evidence supporting nosocomial transmission of a *C. krusei *clone between patients and health care workers although genotyping of small numbers of *C. krusei *isolates during the outbreaks has not been able to exclude the possibility of nosocomial spread of *C. krusei *clones [12,20,21]. A recent cluster of infections was shown to be caused by clonal spread of a single *C. krusei *strain [4]. Mechanisms of nosocomial spread of *C. krusei*, however, remain unclear.

In the present study, we have analyzed a cluster of cases with *C. krusei *infection or colonization in a haematological unit in which the frequency of confirmed invasive *Candida *infections has been low and *C. krusei *has been a rarity. We have searched for potential modes of nosocomial transmission of *C. krusei *between patients and health care workers, and within the environment. In addition, we have investigated possible clonality of the *C. krusei *by genotyping the isolates collected during the outbreak.

## Methods

### Patients and settings

Oulu University Hospital (OUH) is a tertiary care hospital serving a population of 724600 in Northern Finland. The haematological unit of OUH, with total of 928 admissions in 2005, is responsible for treatment of acute leukaemia within that region. 424 admission were due to haematological malignancies and 504 admissions were general internal medicine cases. Eighteen new acute leukaemia cases were diagnosed in 2005 and seven of them underwent allogeneic bone marrow transplantation. In addition, five cases of acute leukaemia relapses were recognized in 2005 but none of them received an allogeneic transplant. The average length of hospitalization was 10.8 days for haematological patients and 3.6 days for patients with other diagnoses. The ward has 20 beds in 11 rooms including 6 isolation units. The unit is operated by two clinical haematologists, one resident in haematology, and 21 nurses working in shifts. In addition, doctors working in various specialties, physiotherapists, and laboratory personnel, for example, are in frequent contact with the patients in the ward.

### Candida infections and usage of antifungals

An unusually high number of *C. krusei *findings was recorded in the haematological unit in February 2005. Before recognition of the outbreak, confirmed invasive infections caused by *Candida *species were rare in the unit: there were four blood culture positive patients with *C. albicans *and one patient with *C. krusei *during 2000 – 2004. Prophylactic fluconazole (400 mg po) was given during neutropenia to several patients that were being considered for allogeneic bone marrow transplantation. Empiric treatment with amphotericin B (conventional or liposomal) was started typically on the fourth day of persisting neutropenic fever. In order to analyze the possible association between antifungal prophylaxis and occurrence of *C. krusei*, data of exposure to fluconazole use was collected from the medical records. The pearson Chi-Square test was used to analyze the significance of fluconazole exposure.

### Microbiological samples

Weekly rectal and pharyngeal swabs were collected for yeast culture in order to follow *Candida *colonization in patients receiving chemotherapy from April 2005 until the end of November 2005. Hand colonization in patients in which the rectal or pharyngeal swabs were positive for *C. krusei *were sampled for yeasts. Pharyngeal and rectal samples were also collected from the health care workers in the unit in order to determine possible colonization with *C. krusei *or other *Candida *species. Environmental samples were collected from all wet and humid locations that were considered to be potential sources of *C. krusei *colonization in the ward. Shower heads, taps, sinks, manholes, and sink traps were examined by selective cultures for yeast and aerobic bacteria.

### Identification of Candida species

*Candida *species were isolated from the samples on Sabouraud Dextrose agar and identified by standard morphological and biochemical methods. A total of 97 *C. krusei *clones were stored at -80°C in skimmed milk and 35 of them were subcultured on Saboraud agar for molecular typing.

### Molecular typing of the Candida krusei isolates

DNA preparation for CHEF electrophoresis was done with a CHEF Mammalian Genomic DNA Plug kit (Bio-Rad, Hercules, CA) accordingly to the manufacturers instructions. Restriction endonuclease digestion was performed overnight at 50°C in buffer containing 2 U of *Sfi*I. CHEF pulsed-field electrophoresis was performed at 13°C for 24 h at 6 V/cm in a 1,2% SeaKem Gold agarose gel (FMC BioProdutcs); the pulse-times varying from 10 to 90 seconds. The gels were stained with ethidium bromide and cluster analysis was done with BioNumerics v4.5 (Applied Maths) using the Dice coefficient to analyze the similarity of the banding patterns, and the unweighted pair group method using arithmetic averages (UPGMA) for cluster analysis. A similairity cutoff of 90% was used to identify genotypes.

## Results

### Outbreak description and infection control measures taken

In February 2005, an increase in the number of culture-confirmed *C. krusei *infections was recognized in the haematological ward. There were six blood culture positive cases (Table 1, patients 1 – 6) and in addition, six patients had significant *C. krusei *growth in samples obtained from deep locations although the blood cultures were negative (Table 1, patients 7 – 12). In three haematology patients the clinical presentation was consistent with invasive fungal infection and they were colonized by *C. krusei *(Table 1, patients 13 – 15). In seven patients the *C. krusei *findings were considered to represent colonization (Table 1, patients 16 – 22) that had to be taken into account regarding empirical antifungal treatment in the persence of persisting neutropenic fever. After recognition of the potential outbreak, intensive use of alcohol-based hand hygiene (70 % alcohol) was encouraged and consumption of the hygiene products was followed. Weekly surveillance of pharyngeal and rectal colonization of all haematology patients for *C. krusei *was started in April 2005 and colonized patients were cohorted according to the surveillance findings. The weekly surveillance cultures were collected until the end of November 2005. Since then, a high number cultures have been taken from patients in which superficial or deep *Candida *infection was suspected. No new clinical infection or colonization by *C. krusei *has been found.

**Table 1:** The table describes the location of *C. krusei *isolation in patients with either confirmed invasive infection, patients with potentially significant infection, or patients with *C. krusei *colonization.

**Patient**	**sex, age**	**Diagnosis**	***C. krusei *isolation**
1	F 32	AML	BSI
2	M 62	AML	BSI
3	M 71	duodenal carcinoma	BSI
4	F 46	mastocytosis	BSI
5	F 64	abdominal surgery	BSI
6	M 86	CLL	BSI
7	M 53	AML	peritoneum
8	M 22	ALL	peritoneum
9	M 33	AML	oesophagus
10	M 39	AML	kidney, liver
11	F 53	AML	invasive candidiasis in autopsy
12	M 55	AML	invasive candidiasis in autopsy
13	F 62	AML	typhlitis, pneumonia
14	M 67	AML	aortitis, pneumonia
15	M 69	multiple myeloma	severe mucositis
16	F 59	AML	rectum, pharynx
17	F 66	MDS	rectum, pharynx
18	F 59	lymphoma	rectum, pharynx
19	F 70	MDS	rectum, pharynx
20	M 22	toxic reaction*	rectum, pharynx
21	M 21	eosinophilic leukaemia	rectum, pharynx
22	M 65	AML	rectum, pharynx

M, male; F, female; BSI, blood stream infection; AML, acute myeloid leukaemia; ALL, acute lymphatic leukaemia; CLL, chronic lymphatic leukaemia; MDS, myelodysplastic syndrome.

*The toxic reaction refers to bone marrow suppression due to alcohol abuse

### Microbiological findings

All haematological patients (a total of 70) were screened for colonization by *Candida *(Table 2). *C. albicans *was the most common species followed by *C. krusei *which was found from 5.2 to 25.7 % of the patients in pharyngeal or rectal samples. Patients that were positive for *C. krusei *in their pharyngeal or rectal swabs, did not carry *C. krusei *on their hands.

**Table 2:** *Candida *colonization of the haematological patients.

	04/2005 (n = 55)	05/2005 (n = 112)	06/2005 (n = 76)	09/2005 (n = 35)	10/2005 (n = 136)	11/2005 (n = 109)
*Candida albicans*	43.6	54.5	50	62.8	70.6	70.6
*Candida glabrata*	0	0	0	0	1.5	8.3
*Candida krusei*	7.2	11.6	5.2	25.7	19.1	13.8
*Candida tropicalis*	3.6	0.9	0	0	0	0
*Candida norwegiansis*	0	0.9	0	0	0	0
*Candida guillermondii*	0	0	2.6	5.7	0	0
*Candida parapsilosis*	0	0	0	0	2.8	0
*Geotrichum spp*	10.9	8.9	6.6	5.7	5.8	0
*Fusarium spp*	0.2	0.9	0	0	0	0
negative	25.5	42.9	52.6	22.9	21.3	22.0

The table shows percentages of surveillance cultures that were positive for fungal species. The samples were collected during April 2005 (04/2005) to November 2005 (11/2005). Some samples grew several fungal species.

Number of cultures (n) is shown in parenthesis.

A total of 21 health care workers were screened for pharyngeal and rectal *C. krusei *colonization. Fifty-five % of the health care workers were colonized by *C. albicans *in their rectum and 62 % in their pharynx. None of them were colonized by *C. krusei *(Table 3).

**Table 3:** Rectal and pharyngeal samples were collected from health care workers and fungal growth was analyzed.

Species	rectum (n = 20)	pharynx (n = 21)
*Candida albicans*	11	13
*Geotrichum*	1	0
*Saccharomyces cerevisae*	1	0

The table shows the number of health care workers positive for the fungal isolates.

Environmental samples from the haematological ward were collected from wet and humid locations that were considered to be potential sites of *C. krusei *colonization. Very little *C. krusei *contamination was found (Table 4). Two locations (sink traps) in a room that was occupied by a *C. krusei *carrier, grew *C. krusei*. None of the incoming water sources or shower heads were positive for *C. krusei*.

**Table 4:** Culture findings in the enviromental samples collected from wet or humid locations in the ward.

Species	tap/incoming water (n = 62)	sink trap (n = 16)	surface (n = 6)
*Fusarium spp*	23	10	3
*Rhodotorula spp*	11	0	0
*Geotrichum spp*	1	3	1
*Candida albicans*	1	3	1
*Candida parapsilosis*	1	0	0
*Candida krusei*	0	2	0
*Candida tropicalis*	1	0	0
*Aspergillus spp*	1	1	0
*Saccharomyces spp*	0	1	0

The table shows culture findings for incoming water sources, sink traps, or on humid surfaces. Number of samples (n) taken from each location is shown in parenthesis.

### Genotyping of the C. krusei isolates

**Figure 1:**
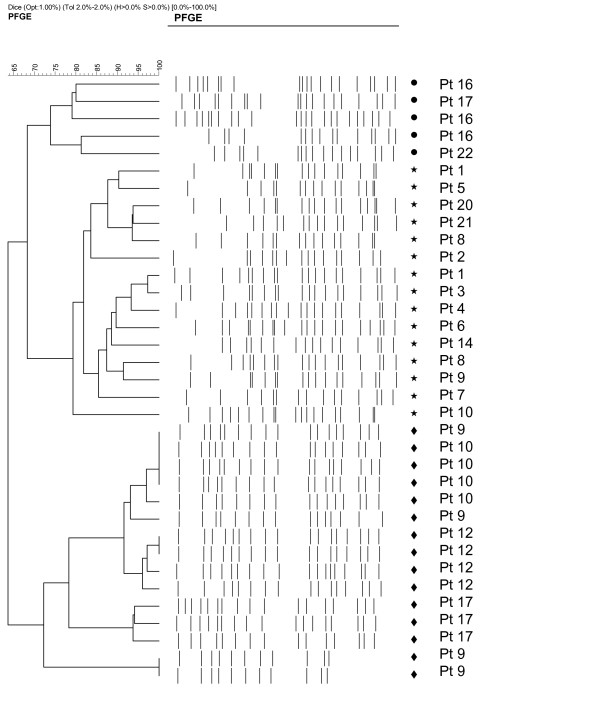
Dendrogram of isolates based on the Dice coefficient, obtained by *Sfi*I macrorestriction analysis of *C. krusei *isolates. The symbols (dot, star, diamond) in the dendogram indicate three large clonal groups identified by PFGE.

A total of 35 *C. krusei *isolates from 18 patients were analyzed by genotyping. This represented single isolations from 12 patients and 23 repeat isolations (2 to 5 isolates from 7 patients each) from clinically significant findings or those isolated from weekly surveillance cultures. Genotyping of these strains showed little evidence of clonal transmission of *C. krusei*. Instead, each patient was colonized or infected by several different closely related genotypes indicated by minor genotypic variation between the repeat isolates collected from individual patients. Some clustering of the genotypes, however, could be obversed (shown in Figure 1). Clones isolated from blood (Patients 1 – 6) were genetically within the same cluster. In addition, using the similarity cut-off of 90%, five groups of patients with similar *C. krusei *strains could be found (patients 1 and 5; patients 20, 21, and 8; patients 1, 3, and 4; patients 8 and 9, patients 9 and 10). Our PFGE typing method showed good discriminatory power and reproducibility between the runs in our study [22].

### Exposure to fluconazole

Fluconazole therapy had been given to twelve of the 22 patients from whom *C. krusei *had been isolated. Eight of the twelve patients, in which *C. krusei *finding was considered clinically significant, were exposed to fluconazole. Eight of the thirteen patients that were treated for acute leukaemia, received fluconazole. During the same time period, nineteen patients with acute leukaemia, that were not colonized by *C. krusei*, was identified and twelve of them received fluconazole. Statistical analysis shows no association between the fluconazole exposure and *C. krusei *in acute leukaemia patients (p = 0.598, Pearson Chi-Square test). In addition, in the haematology patients that were screened for *C. krusei *there was no association with the fluconazole exposure and isolation of *C. krusei *(p = 0.213). Regardless of this, the prophylactic use of fluconazole was stopped and all antifungals were used solely for either empiric treatment of persisting neutropenic fever or proven fungal infections. The patients positive for *C. krusei *received either caspofungin or voriconazole as empiric antifungal treatment.

## Discussion

Invasive infections caused by *C. krusei *has been associated with adverse outcome [23,24]. Poor response to treament may lead to extended hospitalization and economical burden. Therefore, control of emergence of *C. krusei *and other resistant fungal pathogens should be a priorioty. Understanding of risk factors and routes of transmission should help clinicians to prevent infections or to deal with a cluster of infections by *C. krusei*.

It has been shown that cross-contamination of both health care workers and patients by multiple strains of *C. albicans *is possible [25]. *C. parapsilosis *has also been shown to colonize hands of health care workers which may lead to clonal spread of the pathogen to vulnerable patients [19]. Our patients, that were positive for *C. krusei *in their pharyngeal or rectal samples, did not have *C. krusei *growth on their hands. Health care workers at the ward were also screened and *C. krusei *was not isolated from any pharyngeal or rectal samples. In addition, careful analysis of environmental samples for *C. krusei *did not reveal any significant positive results. The incoming water sources were negative for *C. krusei *and we speculate that the *C. krusei *growth in the two sewage samples was an unlikely source of infection. These data do not support the possibile route of clonal transmission by hand contact or from a single environmental source. It must be appreciated, however, that the negative culture results may not rule out occasional exposure of patients or health care workers to *C. krusei *in the ward. Although we were unable demonstrate transmission by hand contact, we continue to emphasize the importance of stringent hand hygiene measures at the ward.

Prophylactic use of fluconazole has been associated with invasive infection or colonization by *C. krusei *although opposing views has been presented [4,11,12,15]. In our study, about half of the patients with acute leukaemia, that were infected or colonized by *C. krusei*, were exposed to fluconazole. The same proportion of leukaemia patients that were negative for *C. krusei*, had been exposed to fluconazole. We have also examined the possible association between the occurrence of *C. krusei *and the use of antifungal agents or drugs used for the treatment of leukaemia. Overall, we were unable to demonstrate any association between the medications and occurrence of *C. krusei*. It is possible that higher number of cases might have revealed such a relationship.

In a previous study, molecular typing of *C. krusei *isolates suggested that clonal spread of a single strain caused an outbreak [4]. Genetic analysis of our *C. krusei *isolates from individual patients suggests mainly for an unrelated origin. Genotyping of several sequential isolates from five patients revealed that each patient harboured their own strain or a slightly altered variant throughout the study period (Figure 1). Some clustering of the genotypes, however, could be observed. Interestingly, the *C. krusei *clones isolated from blood cultures (Figure 1, patients 1–6) were within the same genetic cluster suggesting that those strains may share properties with each other. According to genotyping, we conclude that several different *C. krusei *strains are capable of colonizing the intestinal tract that may serve as an origin of the infection [26].

Most of our patients with *C. krusei *invasion had significant injury of mucosa from which the infection was most likely acquired. Surveillance of mucosal colonization by *C. krusei *was continued until no new invasive infections occurred. Thereafter, we have not found any new cases despite of the high number of samples that have been collected whenever fungal infection was suspected. We speculate that cessation of antifungal prophylaxis with fluconazole and an increased awareness of infection control measures, e.g. hand hygiene and cohorting of the patients, may have been beneficial although we were unable to demonstrate the significance of any single measure.

## Conclusion

Our data indicate that several different *C. krusei *isolates may colonize immunocompromized patients. It seems unlikely that the *C. krusei *colonization of the patients would have originated from contamined hospital environment or the health care workers. We speculate that cessation of antifungal prophylaxis and an increased awareness of infection control measures may have been favorable procedures in order to control the outbreak.

## Competing interests

The author(s) declare that they have no competing interests.

## Authors' contributions

TH and PK were responsible for the infection control measures and desing of the study. II and MK carried out the microbiological identification of the *C. krusei *isolates. MS, TS, and PK were responsible for the care of the patients. HH and JVV carried out the genetic analysis of the *C. krusei *isolates. All authors read and approved the final manuscript.

## Pre-publication history

The pre-publication history for this paper can be accessed here:


